# The key amino acids of E protein involved in early flavivirus infection: viral entry

**DOI:** 10.1186/s12985-021-01611-2

**Published:** 2021-07-03

**Authors:** Tao Hu, Zhen Wu, Shaoxiong Wu, Shun Chen, Anchun Cheng

**Affiliations:** 1 Research Center of Avian Disease, College of Veterinary Medicine, Sichuan Agricultural University, Wenjiang District, Chengdu, 611130 Sichuan China; 2 Institute of Preventive Veterinary Medicine, College of Veterinary Medicine, Sichuan Agricultural University, Wenjiang District, Chengdu, 611130 Sichuan China; 3 Key Laboratory of Animal Disease and Human Health of Sichuan Province, Wenjiang District, Chengdu, 611130 Sichuan China

**Keywords:** Flavivirus, Envelope protein, Key amino acids, Viral attachment, Viral entry

## Abstract

Flaviviruses are enveloped viruses that infect multiple hosts. Envelope proteins are the outermost proteins in the structure of flaviviruses and mediate viral infection. Studies indicate that flaviviruses mainly use envelope proteins to bind to cell attachment receptors and endocytic receptors for the entry step. Here, we present current findings regarding key envelope protein amino acids that participate in the flavivirus early infection process. Among these sites, most are located in special positions of the protein structure, such as the α-helix in the stem region and the hinge region between domains I and II, motifs that potentially affect the interaction between different domains. Some of these sites are located in positions involved in conformational changes in envelope proteins. In summary, we summarize and discuss the key envelope protein residues that affect the entry process of flaviviruses, including the process of their discovery and the mechanisms that affect early infection.

## Introduction

The *Flavivirus* genus, a large genus of important global pathogens, includes broadly distributed human and animal pathogens such as Zika virus (ZIKV), West Nile virus (WNV), Japanese encephalitis virus (JEV), dengue virus (DENV), yellow fever virus (YFV), and tick-borne encephalitis virus (TBEV). Flaviviruses share similar genomic organization and replication patterns and can cause symptoms ranging from flu-like symptoms to severely fatal symptoms. With respect to disease impact, several flaviviruses are neurovirulent and cause central nervous system damage [1, 2], and some member proteins cause increased vascular leakage in a tissue-dependent manner [3], hemorrhage or encephalitis [4]. Flaviviruses pose a major health and economic burden to countries with infected populations [5–7]. In addition, concerns about the potential introduction of these pathogens into new environments, together with the severity of the diseases, have led to the need for further and deeper study of flaviviruses.

**Fig. 1:**
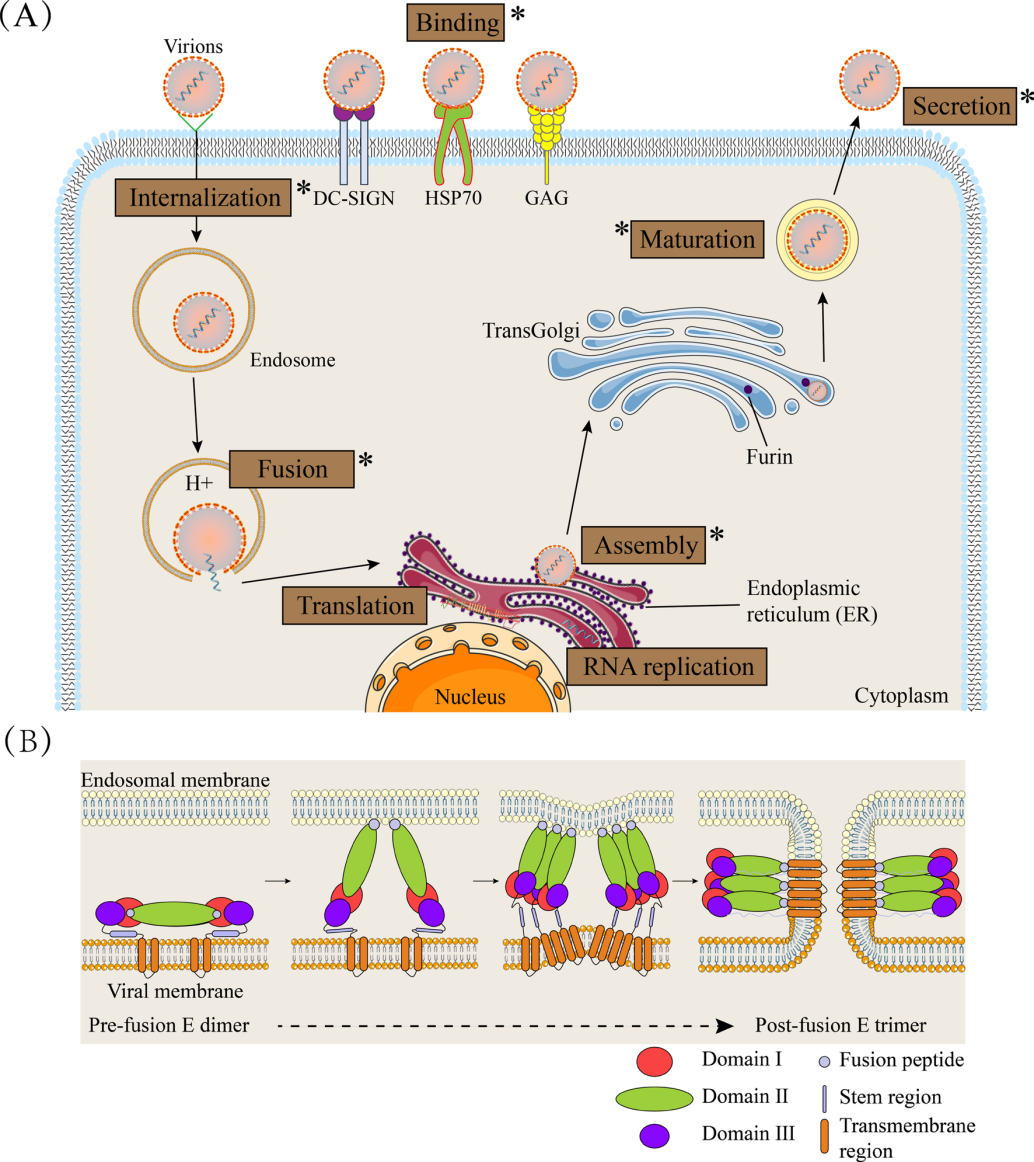
The flavivirus replication cycle and the fusogenic conformational change in the E protein during cell entry. (**A**) Viral particles first interact with attachment factors that are required to bind the virion to the cell surface, which is followed by specific interactions with entry receptors. The attachment factors include DC-SIGN, HSP70, GAG, etc. Flaviviruses enter cells mainly through the clathrin-mediated endocytic pathway. In the low-pH environment of the endosome, conformational changes and rearrangements of the E protein of the virus are triggered that allow the fusion of viral and endosomal membranes, resulting in the release of viral RNA into the cytoplasm. The released positive-sense RNA ((+) RNA) initiates translation at the rough ER membrane and produces a single polyprotein. NS2B3 and cellular signal peptidases cleave the co- and posttranslational polyproteins into structural and nonstructural proteins. Nonstructural proteins participate in RNA replication in the replication complex (RC). (+) RNA can be incorporated into viral particles, which occur in the ER. Following the viral assembly step, the maturation of virions containing prM occurs along the release pathway by furin-mediated cleavage of prM. Mature virions are released by exocytosis. The asterisk indicates the lifecycle in which the E protein participates. (**B**) Schematic of the fusion process. The E dimer anchored in the viral membrane (first panel). The E dimer is separated under the low-pH conditions in endosomes; the fusion peptide is inserted in the endosomal membrane (second panel). Domain III shifts and rotates to create trimer contacts, causing the C-terminal portion of the E protein to fold back towards the fusion loop. The energy released by this refolding causes the membrane to bend (third panel). Generation of the final postfusion structure and opening of the fusion pore (fourth panel). This conformation enables the viral genome to be released into the cytoplasm

**Fig. 2:**
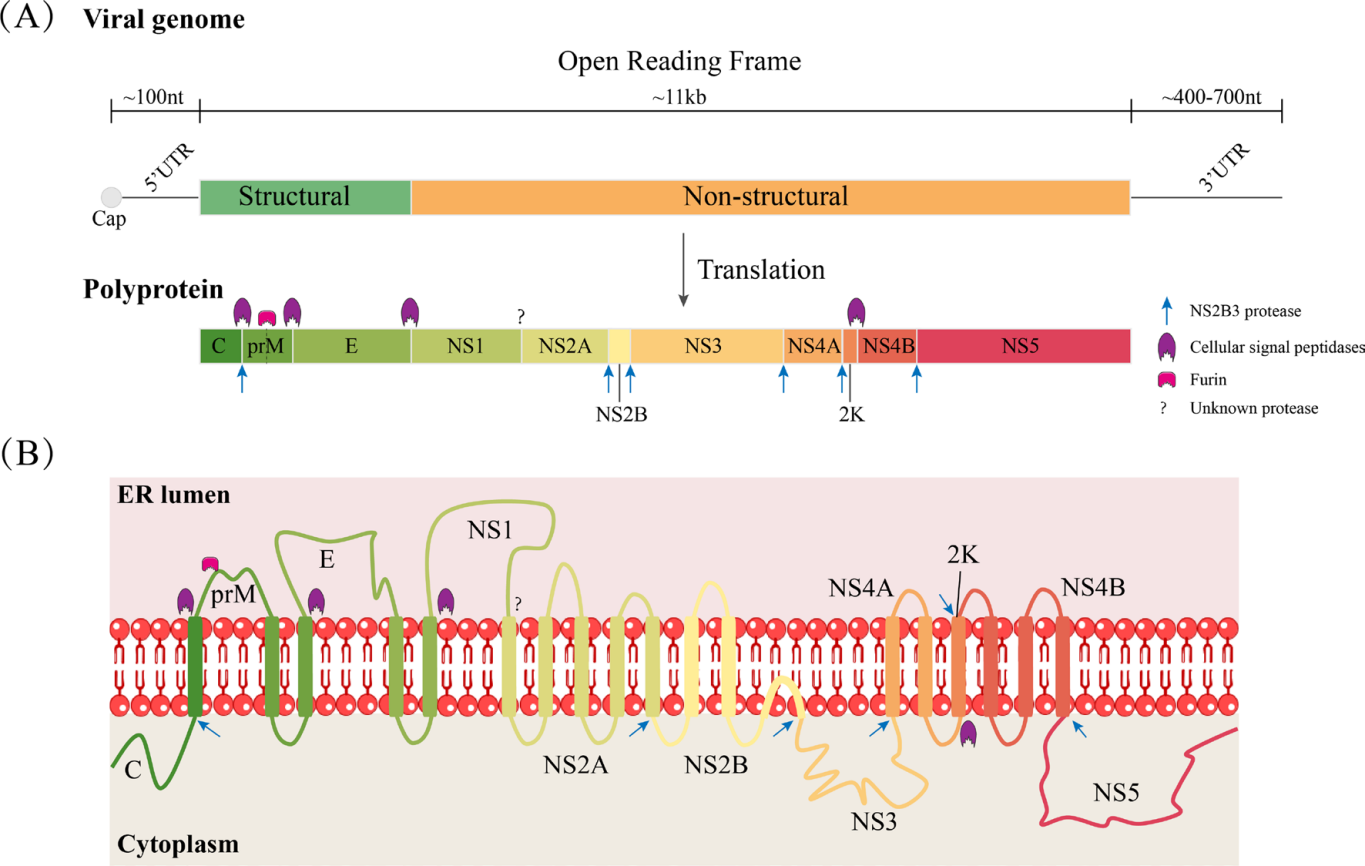
Flavivirus genome organization and membrane topology of mature viral proteins. (**A**) The genome of flaviviruses. Flaviviruses have a positive-sense (+) RNA genome of approximately 11 kb, which has a cap at the 5’ end. The genome of flaviviruses encodes three structural and seven nonstructural proteins that are translated from a single ORF. 5′ and 3′ UTRs are important for translation and RNA synthesis. Polyprotein cleavage by cellular signal peptidases is indicated by purple marks. Blue arrows denote cleavage by the viral protease NS3 and its cofactor NS2B, whereas the pink mark indicates cleavage by the furin protease. The question mark indicates that NS1 and NS2A are cleaved by an unknown protease. (**B**) Polyprotein topology and transmembrane domains of flaviviruses. Flavivirus polyprotein is integrated into the ER membrane. The viral proteins prM, E, and NS1 are mainly on the ER luminal side, and C, NS3 and NS5 are mainly on the cytoplasmic side. Proteins NS2A, NS2B NS4A and NS4B have several transmembrane regions spanning across the ER

Flavivirus infection of host cells is a multistep process. The virus goes through a complex lifecycle to complete the replication and proliferation of the flavivirus (Fig. 1A). The first step of the lifecycle is viral binding and entry. Several cell surface molecules mediate this step [8]. Flaviviruses can utilize different receptors for different cell types and hosts [9, 10]. Following the entry step, flaviviruses are internalized via endocytosis pathways at low pH; then, viral nucleocapsids are released into the cytoplasm (Fig. 1B) [11]. The viral genome in the cytoplasm is used for the synthesis of polyproteins, which are processed by viral and host proteins (Fig. 2A). Genomic RNA replicates in the replication complex within a rearranged endoplasmic reticulum (ER)-derived membrane vesicle (Fig. 2B). When genomic RNA and polyproteins (C, prM and E) are synthesized, they are assembled in the lumen of the ER and processed into immature virions. Subsequently, the immature virions are transported to the trans-Golgi network (TGN) via a secretory pathway for reprocessing. In this step, the prM protein is processed into mature M by furin. Mature virions are released by exocytosis [12, 13].

According to the structure and functions, flavivirus envelope protein (E protein) monomers are divided into three domains (domain I, domain II and domain III) and two regions (stem region and transmembrane region). Domain I participates in E protein conformational changes and stability [14]. Domain II contributes to virus-mediated membrane fusion and contains cross-reactive epitopes and NAb epitopes [15]. Domain III contains linear antigenic epitopes, is used as an antigen [16, 17], and involves E protein stability [18]. The stem region and transmembrane region are involved in virion assembly and affect the prM-E interaction [19].

## Flavivirus envelope protein structure and its functions

**Fig. 3:**
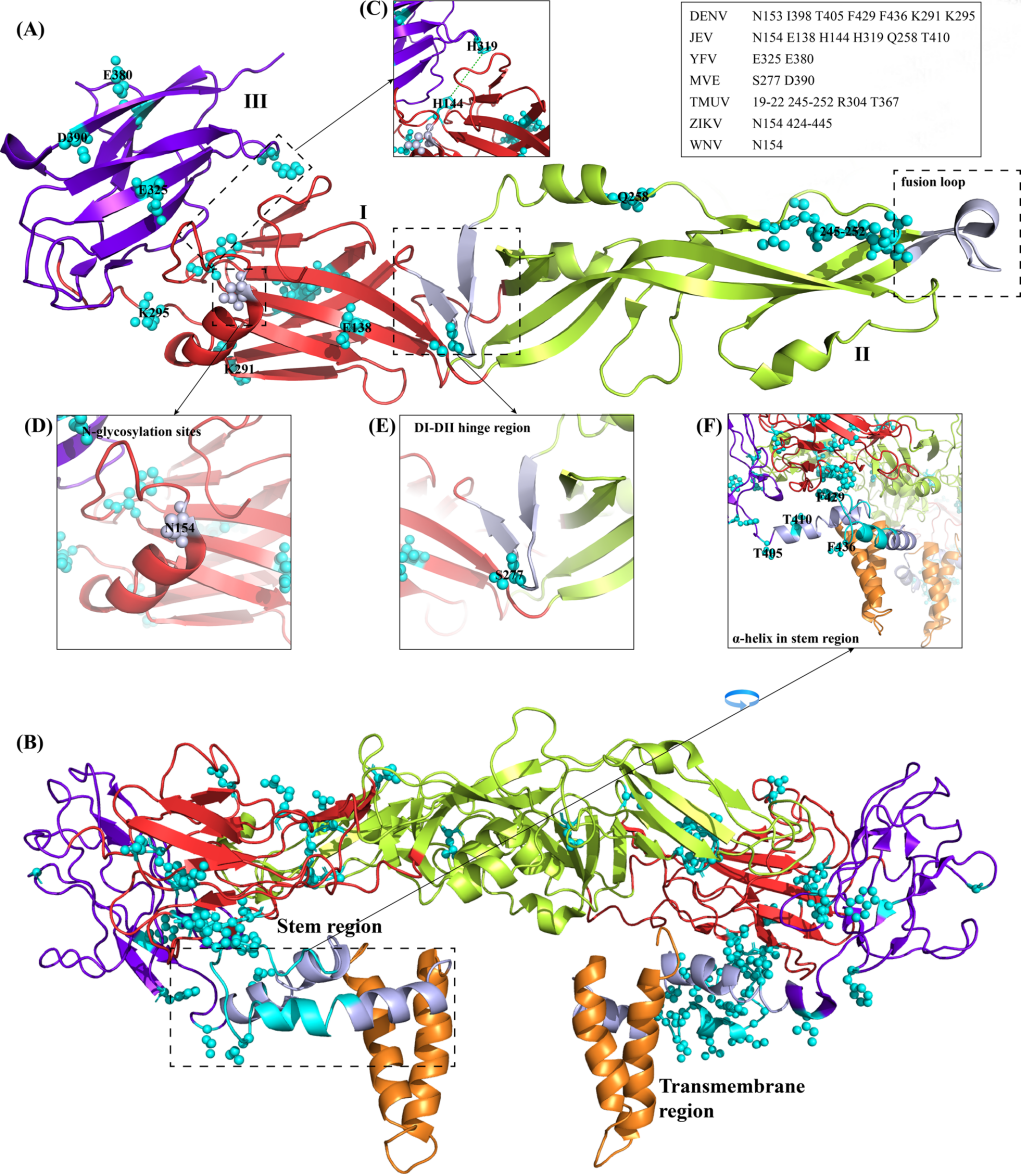
Modeled structure, domain architecture, special structure and mutation sites of envelope protein. Flavivirus E protein diagram representation crystal structure is shown above. Domain I is highlighted in red, domain II is highlighted in limon, domain III is highlighted in purpleblue, stem region is highlighted in gray and transmembrane region is highlighted in orange. Amino acids involved in the early infection process are colored in blue and shown as spheres. The special structure is highlighted in light gray. (**A**) Top view of the Envelope protein monomer structure, JEV (PDB: 5MV1) as the model template [53]. (**B**) Side view of the E dimer structure, ZIKV (PDB: 5IZ7) as the model template. (**C**) The interaction between H144 and H319 is indicated by dashed lines. (**D**) N154 glycosylation site. (**E**) DI-DIII hinge region. (**F**) α-helix in stem region

Flaviviruses are enveloped viruses containing an RNA genome of approximately 11 kb compounded with a capsid protein and surrounded by an icosahedral shell consisting of both the envelope glycoprotein and the membrane or precursor membrane protein anchored in a lipid membrane. On the surface of the mature virion, E is an antiparallel dimer with a fusion loop (Fig. 3A), and the dimer is connected by domain II and domain III. The E protein peptide chain folds into three distinct domains: a central β-barrel (domain I, DI), an elongated finger-like dimerization region (domain II, DII) that includes a fusion loop and is highly conserved in flaviviruses, and an immunoglobulin-like β-barrel structure (domain III, DIII) that is exposed on the viral surface and contains cellular-binding motifs [20, 21]. The C-terminus of DIII is a stem region that contains two α-helices (EH1 & EH2) and a conserved sequence (CS) between EH1 and EH2 (Fig. 3E). The stem region contains two transmembrane helix (TM1 & TM2) regions (Fig. 3B), which are involved in E protein retention in the ER, the processing and location of NS1, and the viral lifecycle [22–24]. The E protein also contains one or two glycosylated asparagine residues that are involved in the interaction between the cell surface and attachment factors [25].

The multifunctional glycosylated E protein is a prototypical class II fusion protein that is an integral part of the virion, participates in viral virulence and virion morphogenesis, and stimulates the production of NAbs [26, 27]. One study showed that the specific motif VNDT (containing an N-linked glycosylation) in ZIKV is involved in mouse neuroinvasion [28]. A culture-adapted TBEV with decreased invasiveness showed a single mutation (D483G) in the E protein, revealing the crucial role of the E protein in viral virulence [29]. In virion morphogenesis, proper folding of the E protein is necessary for prM-E cosynthesis [30], and the expression of E is crucial for the cleavage of the N-terminal signal sequence of the prM protein [31]. As the viral antigen, flavivirus E protein contains many neutralizing antibody targets, which exist in three distinct domains; therefore, the E protein is primarily used as a target for drug therapy [32–34].

Although the E protein is not involved in the replication of genomic RNA, it is responsible for the formation of virions in different lifecycle steps (Fig. 1A, asterisk). In the beginning of flavivirus infection, the E protein serves as the primary bridge to complete host-virus interactions, also participating in membrane fusion and virion uncoating [35]. When the structural protein is translated, the structural protein and the newly replicating viral genome work together in subsequent assembly and release steps [36]. The first step of viral infection of host cells or viral recognition by target cells depends on the interaction between the viral surface and the cellular plasma membrane [8]. In general, cell surface attachment factors are responsible for contact with viral glycoproteins, but their binding is not specific. Each virion can attach one or more factors, such as heparan sulfate and Annexin II [37–39]. This attachment step concentrates virions on the cell surface and facilitates specific interactions between the E protein and entry factors. After the viral particles are combined with the cell surface, the viral particles enter the cell through the endocytic pathway. Once inside the endosome, the viral E protein undergoes low-pH-induced conformational changes, triggering the fusion of host endosomal membranes and viral membranes (Fig. 1B) [40, 41]. Following membrane fusion, viral RNA is released into the cytoplasm.

## The key amino acids of the E protein involved in flavivirus early infection

The E protein is essential for multiple steps of infection and is structurally located on the outermost side of the virion [42]. Substitutions in the amino acids of the E protein may alter the conformation of the flavivirus in various stages of its lifecycle, such as binding, entry, assembly, or release. The change in conformation can be represented as attachment or entry obstacle/enhancement. Changes in these two processes may result in a strength/decrease in the infectivity of the virus or in binding to certain cell receptors (Fig. 3, Tables 1 and 2) [43].

**Table 1:** Summary of the effects of amino acids on virus attachment/entry

Amino acids	Amino acid localization	Structural position/functional site	Viruses	References
154 (DENV, 153)	Domain I	Glycosylation site	DENV, WNV, JEV, ZIKV	[44–47]
398, 405, 429, 436	Stem region	α-helix in stem region	DENV	[48]
424–445	Stem region	Stem region	ZIKA	[49]
138	Domain I	Virulence attenuated site	JEV	[50–53]
390	Domain III	RGD motif	MVE	[54]
277	Between domain I & domain II	Hinger region	MVE	[55]
325, 380	Domain III	Top layer of virion	YFV	[56, 57]
19–22	Domain I	HspA9 binding motif	TMUV	[58]
245–252	Domain II			
304	Domain III	Virulence attenuated site	TMUV	[59]
367	Domain III	Virulence attenuated site	TMUV	[60]
144	Domain I	Participating domain I-domain III interaction	JEV	[61]
319	Domain III			
258	Domain II	Potentially participating post-fusion step		
410	Domain III			
291	Domain III	Participate in electrostatically mediated interactions	DENV	[62]
295				

The table counts the amino acids known to affect the early life cycle of flavivirus E protein. Meanwhile, the structural positions and potential functions of these amino acids are also displayed

**Table 2:** Summary of putative receptors for flavivirus

Molecule	Cells	Viruses	References
DC-SIGN	THP-1	DENV1-4	[123]
Heparin sulfate	BHK21, DEF	TMUV	[38]
Heparan sulfate	Vero, CHO	DENV2	[37]
Hsp70	Huh-7, HepG2	DENV2	[124, 125]
Hsp70	Huh7.5	ZIKV	[126]
Hsp70	Neuro2a	JEV	[127]
GRP78	Neuro2a, Huh7	JEV	[128]
GRP78	HepG2	DENV2	[129]
Hsp90β	Vero	JEV	[130]
Hsp90	HepG2	DENV2	[125]
HspA9	DF-1	TMUV	[82]
NKp44	NK	WNV	[131]
Integrin α_v_β_5_	GSC	ZIKV	[132]
TIM-1, TIM-4	CHO	DENV2	[133]
TIM-1	HEK293T, A549	JEV	[134]
Axl	Human Glial Cells	ZIKV	[9]
CD300a	HEK293T, HeLa	DENV1-4, YFV	[135]
α2,3-linked sialic	Huh7	ZIKV	[136]
Mannose receptor	NIH3T3, Monocytes, Macrophages	DENV1-4	[137]
Prohibitin 1/2	SH-SY5Y, CHME-3	DENV3	[138]
37/67-kDa high-affinity laminin receptor	HepG2	DENV1	[139]
PLVAP and GKN3	Neuro2a	JEV	[140]

The table summarized the identified flavivirus putative receptors

### Domain I and Domain II

DC-SIGN is a C-type lectin receptor expressed on antigen-presenting cells and dendritic cells (DCs) [63]. An early study found that primary human DCs and cell lines transfected with DC-SIGN show extensive infection with DENV [64]. Subsequent studies confirmed that DC-SIGN mediates the infection of DCs by DENV and WNV and mediates the infection of mosquito cells by JEV [46, 65]. In DENV, DCs showed no susceptibility to a viral strain containing two mutations in the E protein (N67 & T155), which demonstrated that DENV glycosylation sites are crucial for DC-SIGN-mediated infection [66]. JEV infects human DCs via the interaction between DC-SIGN and E protein glycosylation sites [46]. In general, most flaviviruses have two glycosylation sites. The importance of envelope protein glycosylation in host-virus interactions was represented in a ZIKA study [67]. In systematically studying the glycosylation sites of ZIKV, the depletion of E glycosylation attenuated ZIKV in A129 mice (Fig. 3D). C6/36 cells were incubated with equal amounts of mutant N154Q or wild-type virus, and viral RNA was detected at different time points post infection. The results showed that the N154Q mutation improved ZIKV attachment, assembly, and infectivity in an in vitro study [44]. Raji cells insensitive to DENV were used as an infection model to compare the infectivity of DENV in naive Raji cells and Raji cells stably expressing DC-SIGN (Raji-DC-SIGN cells). Changing the glycosylation site at asparagine-67 (N67Q) decreased the infectivity of Raji-DC-SIGN cells. This result also occurred in DENV and DENV E-N67Q infection of immature DC cells, indicating that the N-linked glycan at position 67 plays a role in the DC-SIGN-mediated DENV entry process [45]. Another study characterized the amino acids (E-152/156/158) surrounding the ZIKV N-glycosylation site to explore the role of the glycosylation motif region in viral infectivity. Unlike glycosylation sites, a role of E-152/156/158 in viral attachment was not demonstrated. However, the author incubated the virus with cells for 1 h at 4 °C to allow viral attachment, and then chloroquine (an agent that inhibits endosome acidification and restricts viral replication through the inhibition of pH-dependent steps) was added for a 2-h period to restrict pH-dependent endocytosis [68] and quantify intracellular viral RNA [69]. The results showed that these residues affected the viral membrane fusion step. Furthermore, to investigate the effect of E-152/156/158 mutations on the conformation of the E protein, the authors expressed either wild-type or mutant E proteins in mammalian cells, and then performed immunoblotting using structure-specific antibodies (4G2: recognizes fusion loop of most flaviviruses). It was found that 4G2 does not recognize the mutant E protein but the wild-type E protein, indicating that the conformation of the E protein will be altered after the E-152/156/158 change [69]. According to the above studies, we can conclude that E protein glycosylation sites in some flaviviruses (such as JEV, DENV, and ZIKV) or E protein neighboring amino acids play an important role in early infection.

ZIKV, DENV, and JEV are human pathogenic flaviviruses, and vaccine development is an effective method to protect people from these pathogens [70]. A common strategy for obtaining a live vaccine is to pass the isolated wild strains in serial passages to generate attenuated strains with mutations in certain residues, and the attenuated strains are candidates for vaccines [71, 72]. Usually, attenuated strains will differ from the wild type in many ways, for example, by influencing the secretion of the virus and decreasing viremia levels and the efficiency of replication in major target organs [60, 73]. JEV attenuated strain SA14-14–2 (JEV SA14-14–2) is a vaccine strain with good protection effectiveness and safety [74, 75]. By comparing the sequences of multiple JEV attenuated strains, researchers confirmed a high frequency of E138 mutation, and a study confirmed that E138 is related to neurovirulence [50, 51]. Further study of E138 revealed that the acidity/alkalinity of E138 has an effect on the binding of the virus to multiple types of neuronal cells. JEV E138 was replaced with aspartic or histidine (especially histidine), giving JEV a better ability to bind to mouse brain primary cells, Neuro-2a cells and SK-N-SH cells. In addition, this research found that when JEV E138 was substituted with an arginine amino acid (E138R), its susceptibility to heparin-treated cells was enhanced, which indirectly suggested that E138 could contribute to the interaction between the virus and cell surface GAGs [52, 53]. The E protein structure of the SA14-14–2 strain was analyzed to explain the influence of E138 on early infection from another perspective. The E138 change triggers the inversion of the residue at position 279, thus hindering the transition of domain I and domain III to domain II when the E protein matures [76]. Moreover, 138 and 279 residues cooperatively altered the fusion activity [76]. These studies explored the role of E138 in infection and its influence on virulence from different perspectives.

Under low-pH conditions, the protonation of histidine is indispensable for membrane fusion [77]. The key histidine 323 of TBEV functions as a pH sensor in this process, and histidine residues 248, 287 and 323 play a role in stabilizing the structure of the E protein trimer after fusion [61]. During the process of the conformational change of the envelope protein from dimer to trimer, the interaction of domain I and domain III is supported by some conserved amino acids, such as H144 in domain I and H319 in domain III (Fig. 3C) [21]. In JEV, the destruction of these two residues resulted in a significant decrease in the entry activity of the virus [78]. The most likely cause of this result is that the mutation affects the viral membrane fusion process [78].

The hinge region is a linker of domain I and domain II (Fig. 3E), and it has been identified to be the epitope of multiple flavivirus NAbs; because of the specificity of its structure, the hinge region is thought to be associated with entry [79, 80]. Moreover, the hinge region was considered to be relevant to neurovirulence in mice and monkeys in a chimeric vaccine study [26]. The effects of the substitution of amino acids at E277 on different attributes of Murray Valley encephalitis virus (MVE) showed that substitution at this residue had an effect on viral growth kinetics. Further analysis of phenotypes showed that substitution at E277 with different AAs had no significant effect on the binding of the virus to Vero cells. However, hydrophobic AA substitutions at E277 caused a complete (serine to isoleucine, S → I) or marked (serine to valine, S → V; serine to proline, S → P) loss of HA activity, and the HA assay serves as a measure of the ability of viruses to fuse with the host cell membrane [55]. Therefore, compared to mutations that change viral binding ability, the more likely reason is that the mutation disrupts the stability of the E protein β-turn structure near E277.

By establishing the crystal structure of the virus, researchers can actively study sites among E proteins. In the TBEV crystal structure, Q260 and T406 (Q258 and T410 in JEV) form a hydrogen bond at the beginning of the α-B helix of domain II [81]. In addition, JEV Q258 and T410 are considered to potentially participate in the zippering reaction in the postfusion conformation. Alanine mutations at these sites affect viral entry activity [61]. Thus, a disruption of the nature of the two amino acids that form the hydrogen bond leads to a change in viral entry activity [61].

Although the study of site-directed mutations can identify sites that affect early infection, from the viral perspective, it is difficult to find a relationship with specific receptors. Understanding the position of the E protein that binds to the receptors is conducive to targeted intervention for viral infection. Given this, researchers directly analyzed the binding region of the E protein using short peptides synthesized in vitro. HspA9 is a member of the Hsp70 family, and it is reported to be an attachment factor of TMUV (Tembusu virus, an avian flavivirus) [82]. By expressing three domain proteins and performing a coimmunoprecipitation assay, researchers positioned the binding determinants of HspA9 at domain I and domain II, further shortening the length of the peptides, and finally determined that two short peptides (19–22 in domain I and residues 245–252 in domain II) were the key motifs for binding [58].

### Domain III

The crystal structure of the flavivirus E protein revealed that domain III contains four loops, and two (the DE and FG loops) of them are exposed on the viral surface [83]. In a study of these two external loop structures, BHK21 cells were infected with JEV after preincubation with DE loop peptides, and the results showed that the DE loop can inhibit JEV attachment to BHK21 cells [84]. Using a similar method to study the FG loop, the results show that the FG loop has the ability to prevent DENV2 binding to C6/36 cells [85]. For most mosquito-borne flaviviruses, the domain III FG loop contains an Arg-Gly-Asp (RGD) motif that is related to virulence [86, 87]. The RGD motif of many viruses, including rotavirus, hantaviruses and WNV, binds to integrins (heterodimeric transmembrane proteins that consist of α and β subunits and mediate adhesion to the extracellular matrix and cell–cell contact) on the cell surface [88–90]. Researchers characterized the MVE RGD motif by inducing mutations in infectious clones. This study found that the replacement of Asp390 with histidine showed better entry capacity into SW13 cells [54]. In addition, heparin sulfate has been identified as an attachment factor on various flaviviruses [37, 91, 92]. By comparing the sensitivities of different mutant variants to heparin sulfate inhibition of viral attachment, it was found that the glycine mutation exhibited more inhibition sensitivity in Vero, SW13 and BHK-21 cells, and this result showed that E390 is related to viral attachment [54].

Flavivirus E protein domain III is considered to be a receptor binding region [93]. Some studies on vaccine strains have focused on amino acid changes in this area to explore the impact on viral phenotypes [94], such as the YFV 17D strain [95]. A complicated passaging process was required for the acquisition of YFV17D, and during this process, changes in 32 amino acids changed the entire viral protein [96]. Among these differences, residues 325 and 380 located in domain III were shown to be related to virulence in mice [56, 57, 97]. Site-directed mutations at residues 325 and 380 of wild-type YFV were used to determine the effect of the mutation site on the binding ability of the virus to attachment factors (GAGs); the two substitutions significantly reduced sensitivity to heparin inhibition, implying a role in viral attachment [98]. Furthermore, most flaviviruses, including the YFV Asibi strain, exhibit clathrin-mediated endocytosis into the cytoplasm, as mentioned above [40, 41, 99]. Interestingly, the 17D strain E protein mutation changed the mechanism of endocytosis, which no longer depends on clathrin but on dynamin [100]. The above studies have shown that the mutation of the E protein of the 17D vaccine strain greatly changes the viral infection process from attachment to endocytosis.

Electrostatic interactions between negatively charged sulfates (such as GAGs) and basic residues on viral proteins are thought to mediate virus-host interactions [101]. In the DENV study, five highly conserved lysine residues in domain III were selected to study the effect of potential electrostatic effects on virus-cell interactions. Researchers introduced alanine mutations at these positions, expressed recombinant domain III proteins and conducted the GAG-binding ELISA. The results showed that the recombinant protein containing K291 or K295 mutations significantly reduced the binding to GAGs. Furthermore, the ability of the two recombinant proteins to bind to Huh7 cells was reduced, but their ability to bind to C6/36 cells did not. These assays demonstrated that the K291 and K295 residues are important for viral binding in human cell lines but not in insect cell lines [62]. The passage of an attenuated strain of TMUV shows that E-304 is very important to the neurovirulence and neuroinvasiveness of TMUV, and the charged condition of this amino acid plays a key role in the binding affinity between the E protein and GAGs; another study found a similar situation at the E-367 residue [59, 60].

### Stem region

When determining the impact of amino acids on the lifecycle of viruses, more can be learned with the choice of the right method. The packaging system is a powerful tool in the study of lifecycle processes, as well as vaccine candidates [102]. In flavivirus packaging systems, flavivirus replicon-containing reporter genes and trans-supplied structural proteins (CprME or prME) generate SRIPs [103]. By modifying the packaging components and infecting the cells with the modified SRIPs, whether these changes affect the attachment or entry process can be confirmed. In addition, the packaging system can also be used to study the interaction between structural proteins, viral assembly and the screening of viral inhibitors [104–106].

In a DENV study, researchers used proline or alanine to scan mutations in the stem region and used the packaging system to study the entry process. DENV2 CprME containing each mutation (I398, T405, F429 and L436) was cotransfected with the replicon into BHK21 cells (Fig. 3F). After excluding the effect of mutations on structural protein expression by Western blotting, the same number of wild-type or mutant SRIPs were infected into a new round of cells, and then the entry activity of different mutant viruses was indirectly explained by comparing the luciferase activity. After four amino acids were mutated to proline, the entry signal level declined [48]. The reason for the decline in entry activities may be due to the introduction of proline destroying the helical structure. An analysis of the WNV E protein structure by cryo-electron microscopy showed that the stem region extended in the early stage of the membrane fusion process, and this conformational change can give the E protein more space to facilitate rearrangement into a trimer [107]. Therefore, residue alteration may destroy this process and then change the entry ability. Corresponding to this area, a peptide from the ZIKV stem region (E424-445) has antiviral activity in vitro, and this finding may indirectly imply the importance of the stem region in viral entry [49].

## Implication of E protein mutation on vaccine development

The NAb produced by the humoral immune response can protect against viral infections in the long term. Central to most vaccination approaches against flavivirus infections is the E protein. The E protein is the major target of NAbs and contains major neutralizing epitopes [7, 108]. In the development of vaccines, reasonable antigen design may allow vaccines to obtain better immunogenicity and/or improve the safety of the vaccine [109].

The recognition of viral particles by NAbs is closely related to the structure of the E protein. The fusion loop epitope is present at domain II, and its amino acid sequence is highly conserved across flaviviruses. Therefore, many flaviviruses could share the fusion loop epitope and be recognized by specific NAbs (such as the 2A10G6 mAb) [20]. However, cross-reactivity may bring potential risks, causing ADE effects in DENV infection [110]. Artificial modification of E protein amino acids within the fusion loop could reduce this cross-reactivity while retaining the immune response [111]. Domain III has several epitopes due to the particularity of its structure (IgG-like domain). The fully exposed epitope in the maturation virion is the LR epitope, which is accessible for the binding of mAbs. Another two epitopes, the C–C’ loop and ABDE sheet region, were identified in the same study [112, 113]. Furthermore, a variety of NAbs that recognize domain III have been identified [114, 115]. In addition to the above epitopes, the E dimer is crucial for membrane fusion, and some mAbs bind to the epitope to inhibit conformational changes [116]. Some mAbs isolated from patients can also recognize epitopes with E monomers or dimers as structural units [117–119].

Since the NAbs produced after flavivirus infection usually recognize the E protein, the design or modification of the E protein to produce NAbs after immunization is a popular strategy for vaccine research. A ZIKV VLP vaccine that displayed only E protein domain III induced high levels of antibodies, and the antibodies were able to neutralize ZIKV without cross-activity with DENV [120]. Another ZIKV vaccine was designed based on the E dimer as the antigen, in which three cysteine mutations at E-107, 264 and 319 were introduced to stabilize the E dimer and to reduce the exposure of the fusion loop epitope [121]. In summary, general studies have shown that NAbs that can recognize the E protein are easy to obtain [122]. Therefore, current research is more focused on providing good antibody protection while reducing adverse cross-reactivity. In addition to designing better vaccine strategies, this goal may be achieved through amino acid modification.

## Conclusions

Despite differences in the sequences encoding viral proteins, flavivirus E protein has a conserved structure and function. For all flaviviruses, the E protein is closely related to antigenicity, pathogenicity, tissue tropism, NAb recognition and so on. In its own lifecycle, the E protein is involved in the early and late steps of viral infection, such as attachment, entry, membrane fusion, assembly and release. Although some domains affecting the viral lifecycle have been identified, more specific locations or residues participating in these processes need to be studied further. By studying vaccine strains or attenuated strains, amino acids that play a crucial role in the attachment/entry process have been found. Furthermore, packaging systems and site-directed mutagenesis can help us actively search for residues that may be involved in early infection. In addition, the emergence and development of cryo-electron microscopy has helped researchers analyze key sequences or residues in the E protein more conveniently and intuitively. The peptide designed by the E protein itself can also help us indirectly verify the region of the E protein that influences the early step of infection. In HCV, amino acids associated with certain cell receptors have been identified. However, in flaviviruses, binding or entry receptors have not been clearly studied and need further investigation, and we summarized the currently studied putative flavivirus receptors in Table 2.

As the most important flavivirus antigen, the E protein has been selected as the target gene in a variety of vaccine development strategies. An in-depth understanding of the E protein can help us achieve better antigen design. At the same time, the establishment and development of the reverse genetic system has produced a variety of new flavivirus vaccine strategies, such as chimeric vaccines, codon pair deoptimization strategies, and specific mutagenesis of viral determinants of virulence. Although these strategies have significant advantages, it must be noted that the safety of these strategies needs to be further evaluated. In the process of vaccine design and validation, the virulence of the vaccine strain determines whether there is the possibility of continued development and the subsequent inoculation dose. Hence, from the point of view of viruses, the determination of the parts related to the attachment/entry process can provide some help for subsequent study of virus-host interactions and vaccine development. Given this, we summarized the E protein amino acids that are known to participate in the entry process.

## Data Availability

Not applicable.

## Abbreviations

ZIKV: Zika virus
WNV: West Nile virus
JEV: Japanese encephalitis virus
DENV: Dengue virus
YFV: Yellow fever virus
TBEV: Tick-borne encephalitis virus
ER: Endoplasmic reticulum
TGN: Trans-Golgi network
E protein: Envelope protein
CS: Conserved sequence
TM: Transmembrane
NS: Non-structural protein
DC-SIGN: Dendritic cell-specific intercellular adhesion molecule-3-grabbing nonintegrin
DCs: Dendritic cells
GAGs: Glycosaminoglycans
MVE: Murray Valley encephalitis virus
Hsp: Heat shock protein
TMUV: Tembusu virus
RGD: Arg-Gly-Asp
SRIPs: Single round infectious particles
GRP78: Glucose-regulated protein 78
PLVAP: Plasmalemma vesicle associated protein
GKN3: Gastrokine3
VLP: Virus-like particles
ADE: Antibody-dependent enhancement
